# Supplementary material for: Relationship between Health Inequalities and Breast Cancer Survival in Mexican Women
Source: Int J Environ Res Public Health. 2023 Mar 30;20(7):5329. doi: 10.3390/ijerph20075329 (PMC10094649; doi:10.3390/ijerph20075329)
Supplement: Supplementary file 1 [file ijerph-20-05329-s001.zip › ijerph-2222263-supplementary.pdf]

International Journal of
Environmental Research and Public Health

MDPI

Supplementary Material:

Table S1. Use of breast cancer screening programs.

**Questionnaire**		
**Do you know what a screening mammogram is?**	Yes	1 point
	No	0 points
**Prior to your diagnosis, did you undergo a mammogram at least once every two years?**	Yes	2 points
	Occasionally	1 point
	No	0 points
**When was the last time you had a mammogram before being diagnosed?**	A year ago	4 points
	Two years ago	3 points
	Three years ago	2 points
	More than three years ago	1 point
